# Trends in radioactive iodine treatment after total thyroidectomy in Italy, 2001–2018

**DOI:** 10.1530/ETJ-23-0051

**Published:** 2023-07-12

**Authors:** Luigino Dal Maso, Daniela Pierannunzio, Silvia Francisci, Angela De Paoli, Federica Toffolutti, Salvatore Vaccarella, Silvia Franceschi, Rossella Elisei, Ugo Fedeli, the DEPTH Working Group

**Affiliations:** 1 Cancer Epidemiology Unit, Centro di Riferimento Oncologico di Aviano (CRO) IRCCS, Aviano, Italy; 2 National Centre for Disease Prevention and Health Promotion, National Institute of Health, Rome, Italy; 3 Epidemiological Department, Azienda Zero, Padova, Italy; 4 Section of Cancer Surveillance, International Agency for Research on Cancer, Lyon, France; 5 Department of Clinical and Experimental Medicine, Unit of Endocrinology, University Hospital of Pisa, Pisa, Italy

**Keywords:** radioactive iodine (RAI), thyroidectomies, thyroid surgery, time trends, Italy

## Abstract

**Objective:**

A decrease in the use of radioactive iodine (RAI) treatment for thyroid cancer has been described in the last decade in the US following subsequent updates of the American Thyroid Association guidelines. By contrast, population-based data from European countries are lacking. The study aims to assess the frequency and long-term trends in the use of RAI in Italy.

**Methods:**

From the Italian national hospital discharge database, the proportion of RAI treatment after total thyroidectomy with thyroid cancer diagnosis has been assessed by sex and age class during 2001–2018.

**Results:**

Throughout the whole study period, RAI was performed after 58% of 149,419 total thyroidectomies. The use of RAI was higher for men and younger patients; it peaked in 2007 (64% in women and 68% in men) and declined thereafter (2018: 46% in women and 53% in men), with a similar pattern observed across all ages and areas.

**Conclusion:**

National data show that in Italy trends in RAI treatment paraleled those observed in the US. Further monitoring of the use of RAI is warranted in Italy, as elsewhere, to assess the impact of international guidelines on real-life clinical management of thyroid cancer.

## Introduction

Radioactive iodine (RAI) treatment may be used after total thyroidectomy (TT) for thyroid cancer for several purposes: remnant ablation to facilitate detection of recurrent disease, adjuvant treatment of subclinical residual tumor, and treatment of known disease (1, 2). In the 1990s, many experts advocated the use of RAI for nearly all patients submitted to TT (3). However, the benefit of RAI use in low-risk diseases has been questioned as it may cause unnecessary morbidity in patients with no evidence to reduce recurrence or mortality. In addition, RAI treatment is the most important contributor to costs in the postoperative and follow-up period (4) and involves many Italian patients long-distance traveling to reach a Nuclear Medicine Department to perform this treatment.

Beginning with the 2006 American Thyroid Association (ATA) guidelines, which were updated in 2009 and 2015, more selective use of RAI has been recommended (5, 6, 7). In 2009, RAI ablation was no longer recommended for tumors smaller than 1 cm; in 2015, the use of RAI was recommended for patients with aggressive histology or vascular invasion (ATA intermediate risk, the recommendation is weak and based on low-quality evidence).

The impact of updated guidelines on clinical practice has been assessed in the United States (US) using cancer registry data or other large national databases (8, 9). Outside the US, population-based data on changes over time in the recourse to RAI after thyroid surgery are limited (4). Notably, ATA guidelines are highly influential worldwide; but in Europe a wider recourse to postoperative RAI, than in the US, has been suggested until the publication of results from randomized clinical trials on RAI in low- and intermediate-risk patients (10).

This study aimed to provide estimates of time trends from 2001 to 2018 in the use of RAI treatment after TT with thyroid cancer diagnosis in Italy by sex, age groups, and area, thus adding population-based information on the management of thyroid carcinoma paraleling changes in guidelines over the last two decades.

## Methods

In Italy, a public welfare system guarantees universal health care. The National Health Service is centrally organized under the Ministry of Health, and it is administered on a regional basis (19 regions and 2 autonomous provinces). Hospitals (public and affiliated private ones) submit their health claims to the regional health authority for refund. These claims are collected in a single national database containing information at the individual level (Hospital Discharges-HD database) (11). Each record refers to a single hospital episode and includes demographic information (age, sex, and residence), clinical information (main discharge diagnosis and up to five secondary discharge diagnoses, main intervention/procedure and up to five secondary interventions/procedures coded according to International Classification of Diseases, 9th revision, Clinical Modification – ICD9-CM), and dates of admission and discharge. Since 1999, the Ministry of Health has applied data quality control procedures and annually publishes reports on completeness.

In Italy, regional and national health authorities are legislated as collectors of personal data for surveillance purposes without explicit individual consent (12). Research ethics committee approval for research involving this database is not required for a descriptive analysis of anonymous aggregate data without any direct or indirect intervention on patients. The data used for the statistical results shown in this paper were only used in an anonymous form, as prescribed by Italian law (12).

Since both TT and RAI treatment are delivered in hospitals and not in outpatient settings in Italy, they can be fully retrieved from the HD database. The database included all hospitalizations that occurred in Italy from January 1, 2001, to December 31, 2019. A standard anonymization process carried out at the national level assigned to each subject a unique code allowing to group multiple hospitalizations of the same patient, without any possibility of retrieving his identity.

We identified all hospital admissions from 2001 to 2018 related to people residing in Italy with both a TT procedure code (ICD-9CM 06.4, 06.50, 06.52) and a thyroid cancer diagnostic code (ICD-9CM 193) (13). Any subsequent admission with mention of RAI (procedure codes 92.28 and 92.29) within 12 months from the surgery discharge date was searched. The proportion of RAI (only the first administration considered) after TT was computed by sex and age group according to the year of surgery. The median time from discharge after TT to admission for RAI was computed in months. Stratification by area was conducted by administrative regions and aggregated by four macro-areas which show different socioeconomic characteristics: North-West (Liguria, Lombardia, Piemonte, Valle d’Aosta), North-East (Emilia-Romagna, Friuli Venezia Giulia, Trentino, Alto Adige, Veneto), Center (Toscana, Umbria, Marche, Lazio), and South and Islands (Abruzzo, Molise, Campania, Puglia, Basilicata, Calabria, Sicilia, Sardegna) (11).

## Results

Between 2001 and 2018, 577,220 TTs were performed in Italy. Out of these, 149,419 (26%) were admitted for thyroid cancer, 112,828 (76%) were women, and 36,591 were men. Table 1 shows that TT with thyroid cancer diagnosis greatly increased in both sexes from 2001–2006 to 2007–2012 (+51% in women and +61% in men), remaining thereafter stable at about 9500 procedures/year, while the number of all TTs decreased in the last examined period, in both sexes. Through the whole study period, 58% of TTs were followed by RAI treatment within 12 months (58% in women and 60% in men); the median time to treatment was 3.3 months, with a tendency toward a reduction throughout the two analyzed decades (from about 4 months in the first period to 3 months in 2013–2018). The recourse to RAI increased from 2001–2006 (57% in women, 59% in men) to 2007–2012 (62% in women, 64% in men) and declined in the last study years (54% in women, 58% in men) (Table 1).

**Table 1:** Distribution of total thyroidectomies (TT), TT for thyroid cancer (TC), and percentage followed by radioactive iodine (RAI) treatment by sex and period. Italy, 2001–2018.

	Women				Men				Women and men
	2001–2006	2007–2012	2013–2018	2001–2018	2001–2006	2007–2012	2013–2018	2001–2018	2001–2018
All TT	142,987	164,817	142,212	**450,016**	36,296	47,148	43,760	**127,204**	**577,220**
TT for TC	28,022	42,260	42,546	**112,828**	8,413	13,578	14,600	**36,591**	**149,419**
RAI after TT for TC (%)	57%	62%	54%	**58%**	59%	64%	58%	**60%**	**58%**
Median time (days) for RAI^a^	115	101	87	**101**	115	101	87	**101**	**101**

^a^After TT for TC.

**Figure 1:**
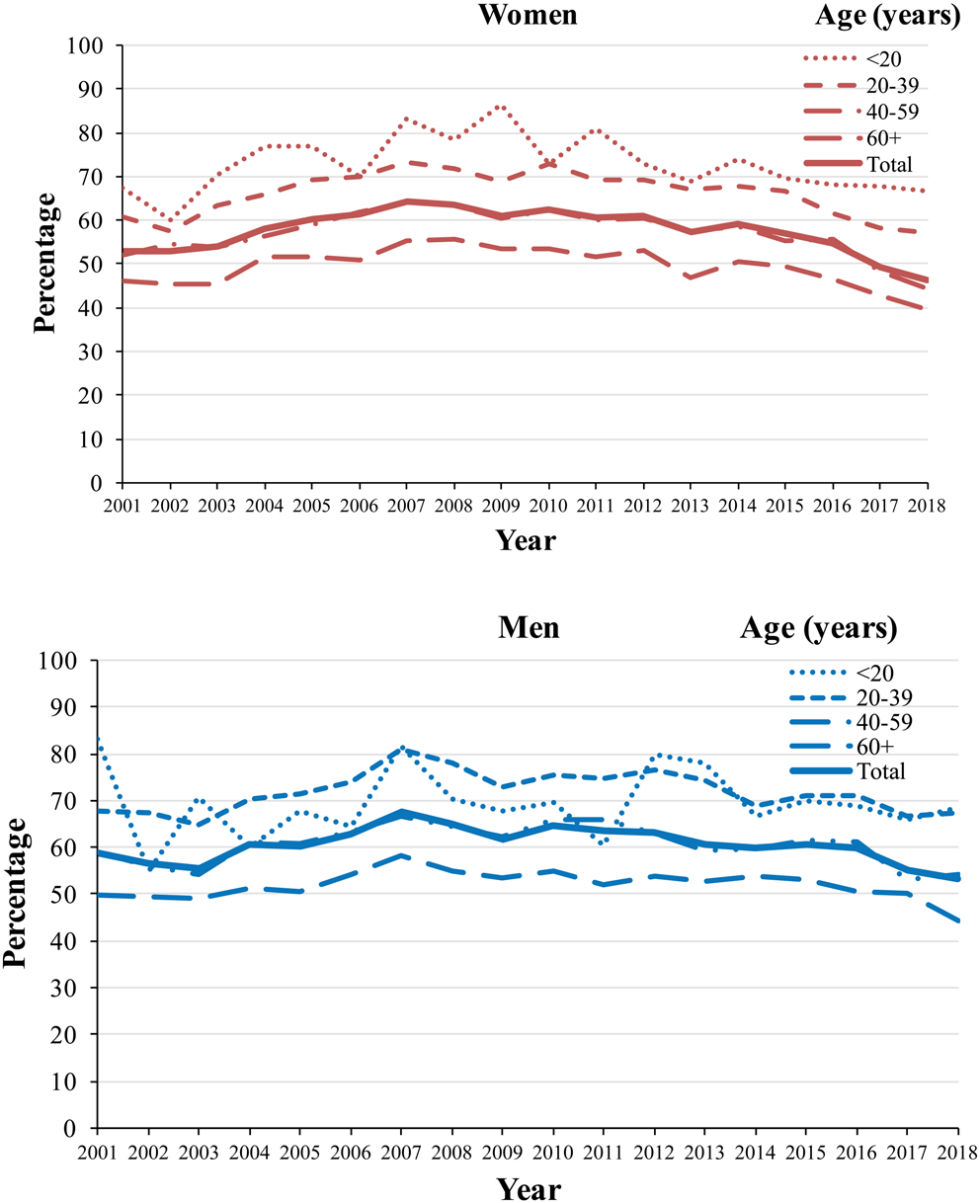
Percent of total thyroidectomies with thyroid cancer diagnosis followed by radioactive iodine treatment by sex and age. Italy, 2001–2018.

**Figure 2:**
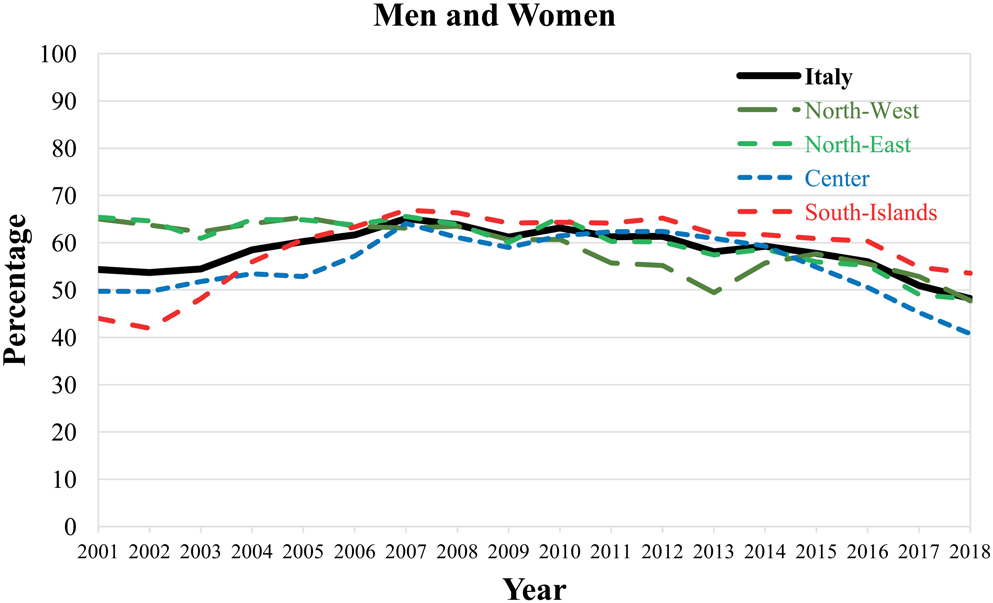
Percent of total thyroidectomies with thyroid cancer diagnosis followed by radioactive iodine treatment by area. Italy, 2001–2018.

Overall, RAI after TT was performed in 53% of women and 59% of men in 2001; such figures peaked in 2007 at 64% and 68%, respectively, and dropped to 46% in women and 53% in men in 2018. Such a time pattern could be observed across all age groups; the proportion of patients treated with RAI was higher in men than women, peaked between 2007 and 2010, and steeply declined in the subsequent years (Fig. 1; numbers on which the percentages were based are reported in Supplementary Table 1, see section on supplementary materials given at the end of this article). Over the whole study period, the recourse to RAI was highest in thyroid cancer patients aged <20 years (>70% in all study periods, based on ~100 per year TT followed by RAI), and decreased in young adults aged 20–39 years (75% in 2007, 59% in 2018), adults aged 40–59 years (65% in 2007, 47% in 2018), and patients aged 60 years or older (56% in 2007, 41% in 2018). Over time, there were geographical variations in RAI use after TT (Fig. 2). In the first study years, RAI treatment was performed in >60% of patients in Northern Italy and <50% in Center and Southern Italy. The recourse of RAI was approximately 60% in all areas from 2007 to 2012 and decreased thereafter, more markedly in Centre (41% in 2018) and Northern (48%), than in Southern Italy (54%).

In the most recent period from 2016 to 2018, when the latest ATA Guidelines were available, a marked decrease was observed in Italy, both in men and in women in the proportion of RAI after TT with thyroid cancer diagnosis corresponding to a nearly 20% decrease in absolute numbers, from 5220 RAI treatments in 2016 to 4219 in 2018 (Supplementary Table 1). Supplementary Table 2 and Supplementary Fig. 1 show incidence rates of RAI after TT per 100,000 women and men in Italy, overall and by age, all descreasing after 2014.

## Discussion

The present study reports population-based data on time trends in the recourse to RAI after TT, rarely reported outside the US. Adoption of RAI in Italy increased in the early 2000s and started to decline after 2007. Nationwide, there were more than 8000 yearly TTs performed with thyroid cancer diagnosis over two decades (9500 per year in 2013–2018). These numbers corresponded to approximately 5240 RAI procedures in Italy during 2013–2018. Notably, our study shows that the use of RAI after TT has decreased during the last decade at all ages, in both sexes and all areas. The decreasing use of RAI was slightly less marked in Italian men and the Southern regions. The Italian Thyroid Cancer Observatory (14) reported use of RAI was generally consistent with the risk-stratified recommendations. However, its frequent use in small (<1 cm) DTCs persists, despite the lack of evidence of benefit.

According to the analyses of the National Cancer Database (NCD), the proportion of patients with well-differentiated thyroid cancer receiving RAI in the US increased from 40% in 1990 to 56% in 2008 (15). Thereafter, a decline in the recourse to RAI for patients with low-risk-differentiated cancer was initially reported from tertiary centers, and subsequently at the population level from the California Cancer Registry (16). In updated analyses of the NCD, among patients with papillary thyroid carcinoma (PTC) submitted to TT, adoption of RAI decreased from 61% in 2004 to 44% in 2016, with a decline beginning in 2007 (9). Using data from 18 Surveillance, Epidemiology, and End Results (SEER) US registries, for PTC <4 cm, the use of thyroidectomy + RAI slightly increased from 38% in 2000 to 44% in 2006, then sharply declined to 18% in 2018; meanwhile thyroidectomy alone and lobectomy increased (8). Similar trends were observed in SEER analyses for PTC ≤1 cm and PTC 1–2 cm (17). Data available from the US differ according to the information source, population coverage, and selection of patients (all well-differentiated thyroid cancers, only PTC, only PTC <4 cm; all selected cancers, or only those submitted TT). However, all reports are consistent with an increase in the recourse to RAI up to 2006–2007, followed by a steep decline.

Outside the US, population-based data are lacking, except for children/young adults (age <20 or <30 years): in Ontario (Canada), no major change could be observed between 1992 and 2010 (18); in South Korea, the recourse to RAI decreased in the last years of observation (19, 20); and in France in 2000–2018, treatment with RAI was administered in above 80% of differentiated pediatric thyroid cancer (21). Only limited insights are available from Brazil where RAI prescriptions increased from 0.45 to 2.28 per 100,000 total inhabitants during 2000–2015 to decrease thereafter (22).

A recent study conducted in France (a country with a healthcare level comparable to Italy's) showed that RAI treatment was administered to about 54% of patients between 2012 and 2016 and contributed substantially to the total diagnostic/therapeutic/care costs of thyroid cancer management (25.6% of total costs) (4).

The main study limitation is the absence of information on histological type, and the staging of thyroid cancer, because these data are not collected in the HD database. In addition, the hospital database included administrative data that are susceptible to coding errors, especially for thyroid cancers that are incidentally discovered after surgery performed for goiter. Sparse available evidence does not show a further increase in the prevalence of incidental papillary microcarcinoma through the last decade in Italy; such prevalence was already high in the mid-2000s according to a multicenter study (23). In addition, other relevant data on histological subtype, post-surgical risk stratification, RAI activity, and preparation for RAI treatment are lacking in the HD database.

Nonetheless, the study demonstrates substantial variations in the frequency of surgical procedures for thyroid diseases in the last two decades, recently reported across Italian regions (11), parallel with relevant changes in the frequency of radioiodine treatment. The trend toward de-escalation of RAI treatment observed in the last decade in Italy was here measured as the proportion of all TT. This trend is unlikely to be a consequence of an increasing proportion of very low-risk differentiated thyroid cancer due to widespread overdiagnosis. Declines in diagnostic pressure, namely fine-needle aspiration biopsies of thyroid nodules, were reported at the population level in some Italian areas after 2014 (24). Meanwhile, the growth in thyroid cancer surgery previously registered in Italy leveled off after 2013–2014 at the national level (11); recent data show a relative increase in the recourse to lobectomy among overall thyroid surgeries in Italy, after a decade of marked decline (24).

In conclusion, the present findings stress the need to monitor RAI treatment in the management of low-risk thyroid cancer in Italy as elsewhere and to assess the impact of international guidelines on real-life clinical management of thyroid cancer, in particular, in younger patients (25), to reduce unnecessary medicalization.

## Supplementary Materials

Supplementary Material

## Declaration of interest

The authors declare no conflict of interest could be perceived as prejudicing the impartiality of the research reported.

## Funding

This work was supported by the Italian Association for Cancer Research (AIRC) (grant no. 21879) and by the Italian Ministry of Health http://dx.doi.org/10.13039/100009647 (RCR-2021-2367121, Alliance Against Cancer, WP 15, PPRER). The funding sources had no involvement in the study design, in the collection, analysis, and interpretation of data, in the writing of the report, and in the decision to submit the article for publication.

## Data availability

Dataset supporting our findings is available, upon reasonable request, by the corresponding authors.

## Author contribution statement

LDM and UF designed the study with the support of DP, S Francisci, ADP, and SFranceschi. DP and S Francisci prepared cleaned data for the study database and did the statistical analyses with the collaboration of ADP, FT, LDM, and UF. SV, S Franceschi, and RE specifically discussed the public health implications of the study results. The first draft of the manuscript was written by UF with the support of LDM. All authors contributed to the interpretation of the study results, reviewed, and approved the final version.
