# Supplementary material for: Trends in radioactive iodine treatment after total thyroidectomy in Italy, 2001–2018
Source: Eur Thyroid J. 2023 Jul 12;12(4):e230051. doi: 10.1530/ETJ-23-0051 (PMC10388682; doi:10.1530/ETJ-23-0051)

Supplementary Table1. Total number of thyroidectomies with thyroid cancer diagnosis followed by radioactive iodine therapy by year, sex, and age. Italy, 2001–2018

		Year																	
**Sex**	**Age (years)**	**2001**	**2002**	**2003**	**2004**	**2005**	**2006**	**2007**	**2008**	**2009**	**2010**	**2011**	**2012**	**2013**	**2014**	**2015**	**2016**	**2017**	**2018**
**Women**	**Total**	**1796**	**2110**	**2446**	**2953**	**3182**	**3517**	**4065**	**4416**	**4357**	**4481**	**4406**	**4497**	**4300**	**4512**	**4215**	**3780**	**3264**	**2990**
	<20	27	30	33	60	70	56	83	72	82	78	85	81	77	86	84	79	69	74
	20-39	583	602	738	868	996	1072	1174	1213	1242	1305	1290	1238	1226	1287	1220	984	914	845
	40-59	755	986	1109	1327	1391	1629	1874	2088	2005	2063	2029	2138	2068	2149	1966	1856	1513	1390
	>=60	431	492	566	698	725	760	934	1043	1028	1035	1002	1040	929	990	945	861	768	681
**Men**	**Total**	**596**	**681**	**728**	**905**	**945**	**1143**	**1329**	**1397**	**1388**	**1502**	**1562**	**1537**	**1559**	**1527**	**1495**	**1440**	**1265**	**1229**
	<20	10	11	17	18	21	18	27	33	25	25	23	36	32	34	21	29	29	24
	20-39	184	185	224	274	256	296	372	404	367	393	414	397	417	368	351	359	302	280
	40-59	249	284	280	391	426	496	583	609	598	673	728	665	665	667	675	643	535	558
	>=60	153	201	207	222	242	333	347	351	398	411	397	439	445	458	448	409	399	367
**Women and men**	**Total**	**2392**	**2791**	**3174**	**3858**	**4127**	**4660**	**5394**	**5813**	**5745**	**5983**	**5968**	**6034**	**5859**	**6039**	**5710**	**5220**	**4529**	**4219**
	<20	37	41	50	78	91	74	110	105	107	103	108	117	109	120	105	108	98	98
	20-39	767	787	962	1142	1252	1368	1546	1617	1609	1698	1704	1635	1643	1655	1571	1343	1216	1125
	40-59	1004	1270	1389	1718	1817	2125	2457	2697	2603	2736	2757	2803	2733	2816	2641	2499	2048	1948
	>=60	584	693	773	920	967	1093	1281	1394	1426	1446	1399	1479	1374	1448	1393	1270	1167	1048

Supplementary Table 2. Rates of thyroidectomies (per 100,000) with thyroid cancer diagnosis followed by radioactive iodine therapy by year, sex, and age. Italy, 2001–2018

		Year																	
**Sex**	**Age (years)**	**2001**	**2002**	**2003**	**2004**	**2005**	**2006**	**2007**	**2008**	**2009**	**2010**	**2011**	**2012**	**2013**	**2014**	**2015**	**2016**	**2017**	**2018**
**Women**	**Total**	5.7	6.7	7.8	9.4	10.1	11.2	13.0	14.2	14.0	14.4	14.1	14.7	14.0	14.4	13.5	12.1	10.5	9.6
	<20	0.5	0.6	0.6	1.1	1.3	1.0	1.5	1.3	1.5	1.4	1.5	1.5	1.4	1.6	1.5	1.5	1.3	1.4
	20-39	6.9	7.2	8.9	10.5	12.2	13.3	14.7	15.3	15.8	16.9	16.8	16.9	17.0	17.9	17.3	14.3	13.6	12.8
	40-59	8.6	11.2	12.6	15.1	15.8	18.6	21.3	23.8	22.8	23.5	22.8	24.1	23.0	23.2	21.0	19.7	16.0	14.7
	>=60	4.8	5.5	6.3	7.8	8.1	8.5	10.4	11.6	11.5	11.5	11.0	11.5	10.2	10.6	10.0	9.1	8.0	7.0
**Men**	**Total**	2.0	2.5	2.6	3.2	3.3	4.0	4.6	4.8	4.8	5.1	5.3	5.3	5.4	5.2	5.1	4.9	4.3	4.2
	<20	0.2	0.2	0.3	0.3	0.4	0.3	0.5	0.6	0.4	0.4	0.4	0.6	0.6	0.6	0.4	0.5	0.5	0.4
	20-39	2.1	2.2	2.7	3.2	3.0	3.6	4.6	5.0	4.6	5.0	5.3	5.4	5.7	5.0	4.9	5.1	4.4	4.1
	40-59	2.7	3.8	3.7	5.1	5.4	6.1	7.0	7.3	7.0	7.8	8.4	7.7	7.6	7.4	7.4	7.0	5.8	6.1
	>=60	2.6	3.3	3.4	3.6	3.8	5.2	5.3	5.3	5.8	5.9	5.6	6.3	6.2	6.2	6.0	5.4	5.2	4.7
**Women and men**	**Total**	3.9	4.7	5.4	6.5	6.9	7.8	9.0	9.7	9.5	9.9	9.8	10.2	9.8	9.9	9.4	8.6	7.5	7.0
	<20	0.3	0.4	0.5	0.7	0.8	0.7	1.0	0.9	0.9	0.9	0.9	1.0	1.0	1.1	0.9	1.0	0.9	0.9
	20-39	4.5	4.7	5.8	6.8	7.5	8.4	9.6	10.1	10.1	10.9	11.0	11.1	11.3	11.4	11.0	9.6	8.9	8.4
	40-59	5.6	7.8	8.5	10.4	10.9	12.6	14.4	15.7	15.1	15.7	15.7	16.1	15.4	15.4	14.3	13.4	11.0	10.5
	>=60	3.9	4.6	5.1	6.1	6.3	7.1	8.3	8.9	9.0	9.1	8.7	9.2	8.4	8.7	8.3	7.5	6.8	6.0

Supplementary Figure 1. Rates of thyroidectomies (per 100,000) with thyroid cancer diagnosis followed by radioactive iodine therapy by year, sex, and age. Italy, 2001–2018

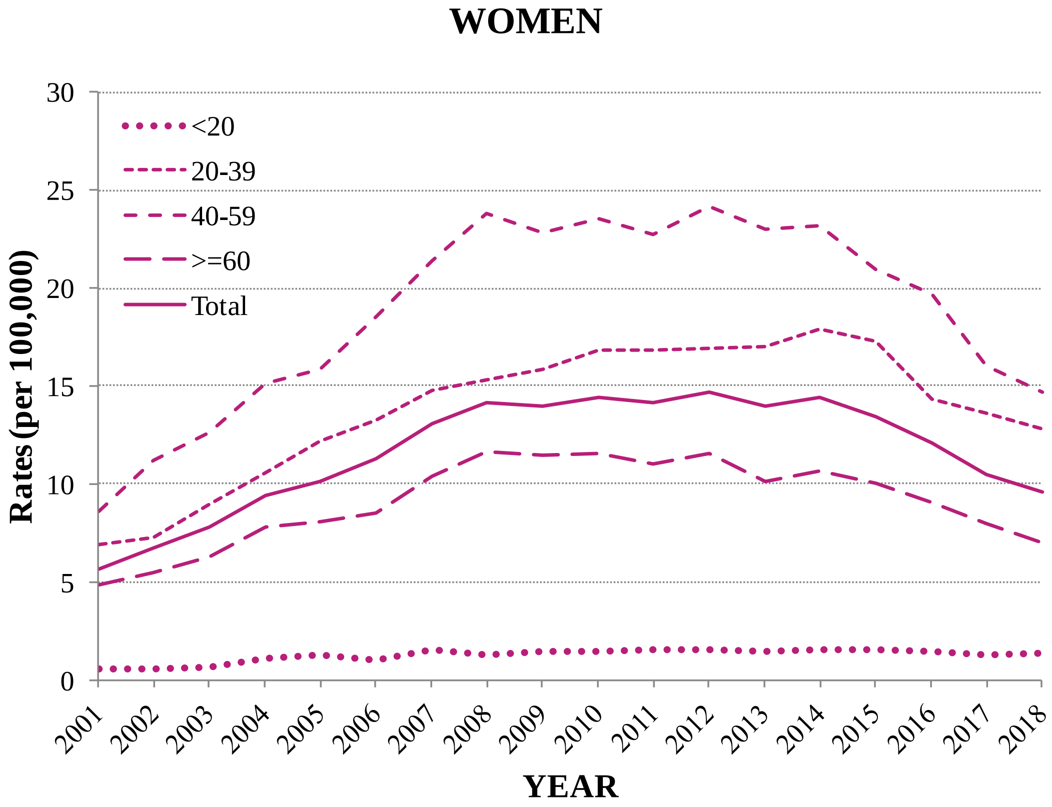

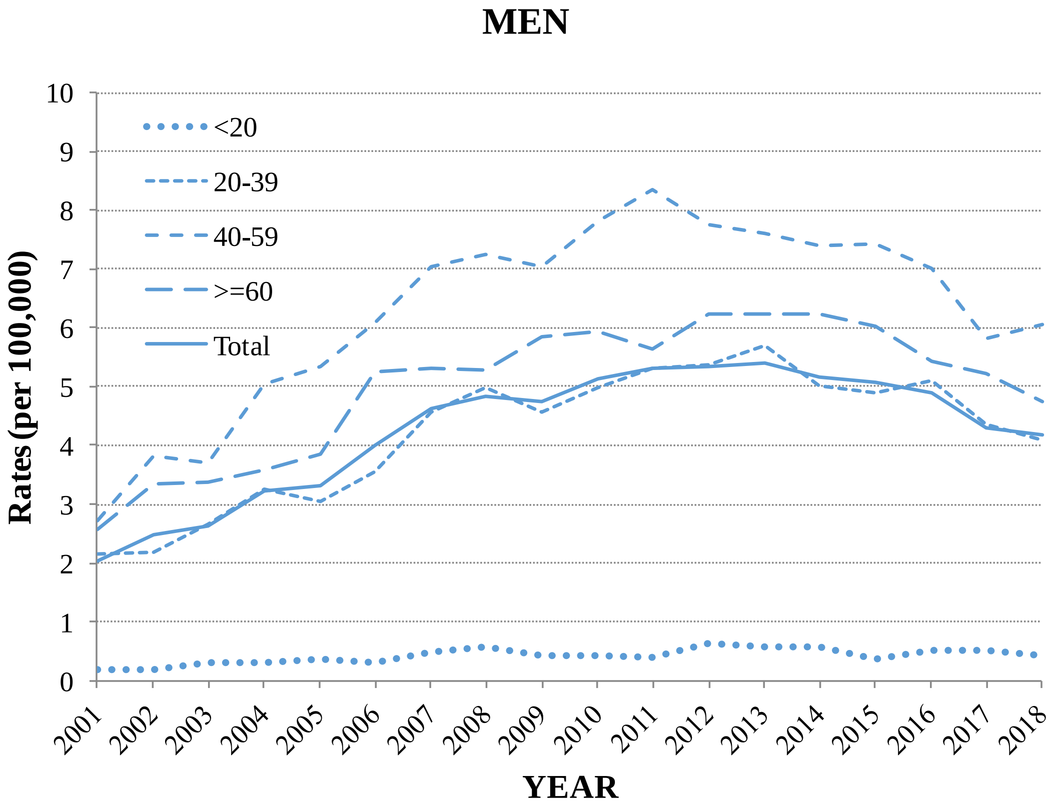

Supplement: Supplementary Material [file supplementary_material.pdf]
